# Measuring Complexity and Predictability of Time Series with Flexible Multiscale Entropy for Sensor Networks

**DOI:** 10.3390/s17040787

**Published:** 2017-04-06

**Authors:** Renjie Zhou, Chen Yang, Jian Wan, Wei Zhang, Bo Guan, Naixue Xiong

**Affiliations:** 1 School of Computer Science and Technology, Hangzhou Dianzi University, Hangzhou 310018, China; renjie_zhou@163.com (R.Z.); yc320719@live.com (C.Y.); magherozhw@hdu.edu.cn (W.Z.); 2 Key Laboratory of Complex Systems Modeling and Simulation of Ministry of Education, Hangzhou Dianzi University, Hangzhou 310018, China; 3 School of Electronic and Information Engineer, Ningbo University of Technology, Ningbo 315211, China; 4 Department of Mathematics and Computer Science, Northeastern State University, Tahlequah, OK 74464, USA

**Keywords:** time series, complexity, sample entropy, flexible similarity criterion, flexible multiscale entropy, sensor network organizing, sensor network controlling

## Abstract

Measurement of time series complexity and predictability is sometimes the cornerstone for proposing solutions to topology and congestion control problems in sensor networks. As a method of measuring time series complexity and predictability, multiscale entropy (MSE) has been widely applied in many fields. However, sample entropy, which is the fundamental component of MSE, measures the similarity of two subsequences of a time series with either zero or one, but without in-between values, which causes sudden changes of entropy values even if the time series embraces small changes. This problem becomes especially severe when the length of time series is getting short. For solving such the problem, we propose flexible multiscale entropy (FMSE), which introduces a novel similarity function measuring the similarity of two subsequences with full-range values from zero to one, and thus increases the reliability and stability of measuring time series complexity. The proposed method is evaluated on both synthetic and real time series, including white noise, 1/f noise and real vibration signals. The evaluation results demonstrate that FMSE has a significant improvement in reliability and stability of measuring complexity of time series, especially when the length of time series is short, compared to MSE and composite multiscale entropy (CMSE). The proposed method FMSE is capable of improving the performance of time series analysis based topology and traffic congestion control techniques.

## 1. Introduction

Time series analysis and forecasting are sometimes essential methods for conquering topology control issues, traffic control issues existed in sensor networks. For example, time series analysis and forecasting is used in optimizing energy-efficient topology organization of WSNs [1,2], in mitigating congestion problem in WSNs [3,4], in detecting faults and anomalies in multi-sensor networks [5,6] and in studying spatiotemporal dynamics of distributed sensor networks [7], and so on. However, one field that has received relatively little attention so far is the measurement of predictability or complexity of time series generated by sensor networks. In our opinion, measurement of time series complexity and predictability is a more fundamental and basic part of the whole solutions to challenges lie in wireless sensor networks. Here, the complexity of time series is regarded as the difficulty in predicting its future patterns.

While several types of entropies for measuring complexity of time series have been extensively studied in the literature, almost all of them are focused solely on the single-symbol properties of time series characteristics [8], which probably leading to the omission of large amount of information. In paper [9], the authors demonstrated that multiscale entropies provide new information about time series. In this paper, we further propose a novel method based on multiscale entropies, which is capable of discriminating the difference between noise and interference in the empirical time series. The proposed method is evaluated on both synthetic and real time series, and all of the results demonstrate that the proposed method has a significant improvement in reliability and stability of measuring complexity and predictability of time series. Our method could be applied in various ways to help researchers conquer the challenges in fields of sensor networks.

The rest of the paper is organized as follows: related works are introduced in Section 2. Section 3 introduces the preliminaries of sample entropy, multiscale entropy and composite multiscale entropy. The flexible multiscale entropy is proposed in Section 4 and evaluated in Section 5. Finally, we conclude the paper in Section 6 and future work is presented in Section 7.

## 2. Related Works

As mentioned in introduction, there have been many works in the literature regarding to applying time series analysis and forecasting methods to conquer challenges in sensor networks organizing, monitoring and anomaly detection. Furthermore, in many cases, the solutions to challenges lie in wireless sensor networks using time series analysis are based on the analysis of time series similarity and complexity. In paper [10], the authors presented a novel anomaly detection method based on analyzing similarities of time series in sensor networks. Kasetty et al. proposed a framework for classification of time series that collected by sensors [11]. Regarding to measuring complexity of time series, several entropy-based metrics have been proposed, e.g., approximate entropy [12] and sample entropy [13]. The two entropy metrics quantify the degree of regularity of a time series by evaluating the occurrence of repetitive patterns. Be different from approximate entropy, sample entropy excludes the case of self-matching. The sample entropy has a higher speed of stability convergence and a less dependency on the length of time series than approximate entropy. Previous works have reported that sample entropy is an effective and efficient method to gain insights into various signals, such as, electroencephalography signals [14] and heart rate time series [15], etc.

However, sample entropy analyzes time series only at a single time scale. Hence, it fails to capture the long-range dependence of a time series. In responses to this problem, Costa proposed multiscale entropy (MSE) to measure the structural complexity of a time series over different time scales [16,17]. Structural complexity refers to “meaningful structural richness” [18], incorporating correlations over multiple spatiotemporal scales. In the context of structural complexity, neither completely predictable signals, nor completely unpredictable signals are truly complex, since they can be described very compactly [17].

MSE has been widely applied in many fields. Costa used it to analyze complexity of biological and physical signals [16,17]. Ge et al. applied MSE theory in electroencephalograph (EEG) signal detection [19], finding that the value of entropy has an obvious change when people stay in different sleep stages and the change of MSE is consistent with the physiological mechanism of brain activity. Zhang et al. studied rolling bearing fault detection with MSE, and proposed a new metric named multiscale entropy mean deviation and applied in bush fault diagnosis and prediction [20]. Xie [21] applied MSE to the analysis of geophysical observation signals, and proposed concepts of local multiscale entropy and generalized entropy spectrum. These theories were applied to various kinds of complicate geographical signal analysis, digging out more signal characteristics and information. The authors of paper [9] applied MSE to the study of network traffic characteristics, and demonstrated that MSE has obvious advantages over information entropy and self-similar parameter. MSE has also been applied to many other aspects such as wireless mobile traffic analysis [22], early Alzheimer’s disease diagnosis and mild cognitive impairment of wave detection [23], electromyography (EMG) signal detection [24], crude oil price [25], mood states modulate complexity [26], soil transect data [27], financial time series [28], rainfall-runoff relationships [29], rotor fault diagnosis [30], etc.

In the coarse-graining process of MSE analysis casted on a time series with N points, only the first coarse-grained time series is used to calculate entropy values. As a result, the number of points decreases from N to N over time scale τ. The problem is that, when the length of the original or the coarse-grained time series is shorter than, for example, 750 points, the deviation of the estimated entropy values raises very quickly as the number of data points decreases. Large deviation of estimated entropy values leads to a considerably reduced reliability and stability in discriminating time series generated by different systems or by the same system under different conditions. Currently, there are several solutions to the problem. One type of solutions is to replace the coarse-graining process. Liu and Wei et al. employed an adaptive resampling procedure to replace the coarse-graining process in MSE, which is able to reduce the variation of entropy values caused by the length limitation of signals [31]. More recently, the authors proposed multivariate empirical mode decomposition enhanced multivariate multiscale entropy (MEMD-enhanced MMSE) to evaluate the balance stability of vibration shoes. The balance stability of shoes is significantly improved under the assistance of MEMD-enhanced MMSE, compared with the original MSE [32]. Wu et al. reported a modified MSE (MMSE) algorithm, which replaces the coarse-graining procedure of MSE with a moving-average procedure [33]. This study showed that the MMSE algorithm is more reliable than the conventional MSE in analysis of short-length time series. Authors in paper [34] applied the modified multiscale entropy (MMSE) to study the computer operating behavior characteristics of human beings and found that retiree group exhibits higher complexity than student group and worker group. Another type of solutions is to improve the coarse-graining procedure. Wu et al. proposed composite multiscale entropy (CMSE) [35] and refined composite multiscale entropy (RCMSE) [36], which significantly reduce the deviation of the estimated entropy values by taking the average of the entropy values of τ coarse-grained time series instead of that of just the first coarse-grained time series. Niu et al. studied the characteristics of stock indices using CMSE and confirmed that CMSE is better than MSE in stability and reliability [37]. They also adopted the CMSE to demonstrate the effectiveness of a financial time series agent-based model proposed by them [38]. CMSE has been applied in analyzing many other materials, such as bistable laminated plate signals [39], and magnetoencephalography recordings, etc. More recently, the composite coarse-graining procedure has also been adopted to reduce the length dependence of multiscale permutation entropy [40,41].

However, sample entropy-based methods, including MSE and CMSE, measure the similarity of two subsequences of a time series with either zero or one, but without in-between values. These methods probably output sudden changes of entropy values even if the time series embraces small differences. This problem becomes especially severe when the length of time series is getting short. With regard to this problem, we propose flexible multiscale entropy that measures the similarity of two subsequences with full-range values from zero to one, and thus decreases the fluctuation of entropy values. In order to demonstrate the effectiveness of the proposed method, we will carry out experiments with both synthetic and real time series.

## 3. Preliminaries

### 3.1. Sample Entropy

Sample entropy is now widely used for measuring the complexity of time series. Sample entropy reflects the conditional probability that two similar sequences of length m remain similar when one more consecutive point is added to each sequence. As an improved method of approximate entropy, sample entropy avoids self-match in the template matching process. This improvement enables sample entropy to reflect the complexity of data sequence more accurately and to be largely independent of time series length [13]. The calculation steps of sample entropy are as follows:

- Step 1: Given a time series containing *N* data points {x(i)|1≤i≤N}, consider m dimensional vector sequences, $X_{m}(i)=[x(i),x(i+1),...,x(i+m-1)],$ 1≤i≤N−m+1, *m* is called pattern length in the rest.
- Step 2: Define $d[X_{m}(i),X_{m}(j)]$ as the distance between the two vectors, which equals the maximum absolute difference between the corresponding elements in the two vectors. The expression is as follow:
  (1) $$d[X_{m}(i),X_{m}(j)]={\left\|X_{m}(i)-X_{m}(j)\right\|}_{\infty }=\max\{|x(i+k)-x(j+k)|\}$$
  where: 0 ≤k≤m−1,1≤i,j≤N−m+1.
- Step 3: Set the similarity criterion *r*, the probability that the other vectors are similar to vector $X_{m}(i)$ is defined as $B_{i}^{m}(r)$:
  (2) $$B_{i}^{m}(r)=\frac{1}{N-m-1}num\{d[X_{m}(i),X_{m}(j)]<r\},\;1\leq i,j\leq N-m,i\neq j$$
  where *num* accumulates the number of similar vectors, which are vectors having a distance to $X_{m}(i)$ smaller than *r*.
- Step 4: Calculate the average value of $B_{i}^{m}(r)$, denoted as $B^{m}(r)$, which indicates the probability that two vectors will match for *m* points:
  (3) $$B^{m}(r)=\frac{1}{N-m}\sum_{i=1}^{N-m}B_{i}^{m}(r)$$
- Step 5: Set pattern length to *m* + 1, calculate $A_{i}^{m}(r)$:
  (4) $$A_{i}^{m}(r)=\frac{1}{N-m-1}num\{d[X_{m+1}(i),X_{m+1}(j)]<r\},1\leq i,j\leq N-m,i\neq j$$
- Step 6: Calculate the average value of $A_{i}^{m}(r)$, denoted as $A^{m}(r)$, which represents the probability that two vectors will match for *m* + 1 points:
  (5) $$A^{m}(r)=\frac{1}{N-m}\sum_{i=1}^{N-m}A_{i}^{m}(r)$$
- Step 7: Calculate sample entropy:
  (6) $$SampEn(m,r)=\underset{N\to \infty }{\lim}\{-\ln\frac{A^{m}(r)}{B^{m}(r)}\}$$

In the actual calculation process, we often use the following formula:

(7) $$SampEn(m,r,N)=-\ln[\frac{A^{m}(r)}{B^{m}(r)}]$$

Conventionally, *m* = 2, *r* = 0.15, which means similarity criterion is 0.15 × *SD*. *SD* is the standard deviation of the original time series. Comparing to approximate entropy, sample entropy has better consistency. In the calculation process of vector matching, no self-match is included. It makes sample entropy more accurate in describing the complexity of time series and makes sample entropy largely independent of data sequence length [13].

### 3.2. Composite Multiscale Entropy

The composite multiscale entropy (CMSE) is proposed in paper [35]. CMSE improved the stability of calculation by using composite averaging method. The effectiveness of the theory is verified on two types of artificial noise signals and a real vibration data set. The “coarse-graining” process of CMSE is different from that of MSE. For every scale factor τ, the given time series {x(i)|1≤i≤N} will be transformed to:

(8) $$y_{k}^{\left(\tau \right)}=\{y_{k,1}^{\left(\tau \right)},y_{k,2}^{\left(\tau \right)},...,y_{k,P}^{\left(\tau \right)}\},P=\left\lfloor \frac{N-k+1}{\tau }\right\rfloor ,\;1\leq k\leq \tau$$

The specific transformation formula is as follow:

(9) $$y_{k,j}^{\left(\tau \right)}=\frac{1}{\tau }\sum_{i=(j-1)\tau +k}^{j\tau +k-1}x_{i},\;1\leq j\leq P,\;1\leq k\leq \tau$$

In the CMSE algorithm, for every scale factor τ, CMSE value is calculated as the mean of sample entropy values:

(10) $$CMSE(x,\tau ,m,r)=\frac{1}{\tau }\sum_{k=1}^{\tau }SampEn(y_{k}^{\left(\tau \right)},m,r)$$

## 4. Flexible Multiscale Entropy

As can be seen from Equation (2), sample entropy measures the similarity of two subsequences with either one or zero. If the distance between two vectors is less than *r*, then the two vectors are regarded as similar, in other words, the similarity of the two vectors is assigned as one. Otherwise, the similarity of the two vectors is zero. Such similarity metric has a problem that the similarity value changes suddenly from one to zero if the distance between two vectors cross the borderline of *r* and vice versa. As a result, entropy values are considerably impacted even if the time series embraces only small differences. This problem becomes especially severe when the length of time series is getting short, e.g., less than 750 points.

To this end, we propose flexible multiscale entropy (FMSE) that measures the similarity of two subsequences with full-range values from zero to one, and thus decreases the fluctuation of entropy values. Before introducing the calculation procedure of FMSE, it is necessary to point out the main difference between the calculations of FMSE and MSE. In the traditional MSE analysis, sample entropy is calculated as shown in Equation (7). In the formula, $B^{m}(r)$ and $A^{m}(r)$ are calculated in the same method as given in Equations (3) and (5). However, in FMSE, the calculation of $A^{m}(r)$ is different from that of sample entropy. In order to distinguish from $A^{m}(r)$, we will define $C^{m}(f)$ in FMSE. The calculation process of FMSE is shown as follows:

- Step 1: Incorporating the idea of composite coarse-graining from the CMSE. For every scale factor τ, we transform the original time series {x(i)|1≤i≤N} to new time series $y_{k}^{\left(\tau \right)}$ as Equations (8) and (9).
- Step 2: For time series $y_{k}^{\left(\tau \right)}$, calculate $B^{m}(r)$ same as Equations (1)–(3). Note that, in the process of calculating $B^{m}(r)$, the length of time series is *P*, since the new time series has been coarse-grained from the original time series.
- Step 3: In this step, calculate $C_{i}^{m}(f)$. Different from the similarity accumulating function in sample entropy, we define a new accumulative function $s(Y_{k,m+1}^{\left(\tau \right)}(i),Y_{k,m+1}^{\left(\tau \right)}(j))$, which is a piecewise function that avoids the similarity of vectors changing suddenly between 0 and 1:
  (11) $$s(Y_{k,m+1}^{\left(\tau \right)}(i),Y_{k,m+1}^{\left(\tau \right)}(j))=\{\begin{array}{cc} 0 & d[Y_{k,m+1}^{\left(\tau \right)}(i),Y_{k,m+1}^{\left(\tau \right)}(j)]\geq f\\ 1-\frac{d[Y_{k,m+1}^{\left(\tau \right)}(i),Y_{k,m+1}^{\left(\tau \right)}(j)]}{f} & d[Y_{k,m+1}^{\left(\tau \right)}(i),Y_{k,m+1}^{\left(\tau \right)}(j)]<f \end{array}$$
  where *f* is called flexible similarity criterion, *f* is a proportion, usually set to 0.2, of standard deviation of the original time series.

The ratio of similar vectors for pattern length *m* + 1 is as follows:

(12) $$C_{i}^{m}(f)=\frac{1}{P-m-1}\sum_{j=1}^{P-m+1}s(Y_{k,m+1}^{\left(\tau \right)}(i),Y_{k,m+1}^{\left(\tau \right)}(j)),1\leq i\leq P-m,i\neq j$$

- Step 4: Calculate $C^{m}(f)$ as follows:
  (13) $$C^{m}(f)=\frac{1}{P-m}\sum_{i=1}^{P-m}C_{i}^{m}(f)$$
- Step 5: Calculate the improved sample entropy for coarse-grained time series $y_{k}^{\left(\tau \right)}$:
  (14) $$FSampEn(y_{k}^{\left(\tau \right)},m,r,f)=-\ln[\frac{C^{m}(f)}{B^{m}(r)}]$$
- Step 6: Calculate flexible multiscale entropy:
  (15) $$FMSE(x,\tau ,m,r,f)=\frac{1}{\tau }\sum_{k=1}^{\tau }FSampEn(y_{k}^{\left(\tau \right)},m,r,f)$$

**Figure 1:**
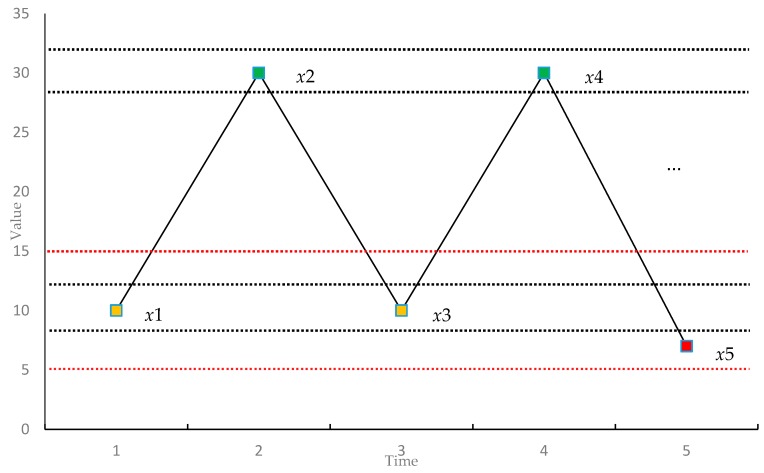
A time series for illustrating the calculation of flexible multiscale entropy.

Figure 1 shows a time series {x(i)|1≤i≤5} for illustrating the calculation process of FMSE. The black dashed lines around x(1) and x(2) represent x(1)±r×sd and x(2)±r×sd, respectively, where *sd* stands for the standard deviation of the time series and *r* is the similarity criterion, which is typically set to be between 0.1 and 0.2. In the following, we take the case when *m* is 1 as the example. In order to compute the FMSE for this case, we need to obtain $B^{1}(r)$ and $C^{1}(f)$. Consider the one-point-pattern for $x_{1}$, we need to find out all the points that match with $x_{1}$, which are the points fall in between the black dashed lines x(1)±r×sd. In this example, $x_{3}$ is the only one point that satisfies the requirement. Then, the value of $B_{1}^{1}(r)$ is 1/3. Similarly, for points $x_{i}\left(2\leq i\leq 4\right)$, the value of $B_{i}^{1}\left(r\right)$ (2≤i≤4) is 1/3, respectively. Thus, the value of $B^{1}(r)$ is 1/3. For computing $C^{1}(f)$, we need to consider the sequences with pattern length of *m* + 1, that is 2 in this case. As shown in Equations (11) and (12), we introduce a flexible factor *f* in the computation of $C^{1}(f)$. The red dashed line shown in Figure 1 is $x_{1}$ ± *f* * *sd*. Consider the two-point-pattern ($x_{1}$, $x_{2}$), we can find that the pattern ($x_{3}$, $x_{4}$) matches with it. In this case, we also find that ($x_{4}$, $x_{5}$) matches with ($x_{2}$, $x_{3}$). The calculation of cumulative number of similar patterns has also been improved in our method. For example, for the pattern ($x_{3}$, $x_{4}$) and ($x_{1}$, $x_{2}$), we measure the similarity as $\left(1-\frac{d\left[\left(x_{1},x_{2}\right),\left(x_{3},x_{4}\right)\right]}{f}\right)$ instead of 1 here. In this way, the method can avoid the similarity of patterns changing suddenly between 0 and 1. After obtaining $C^{1}(f)$, we then compute the FMSE according to Equations (14) and (15).

## 5. Experiment and Evaluation

In this section, the proposed FMSE will be evaluated against MSE and CMSE through two synthetic noise signals and a set of real vibration data collected using sensors by the Case Western Reserve University (CWRU) Bearing Data Center [42].

### 5.1. Synthetic Noise Time Series

**Figure 2:**
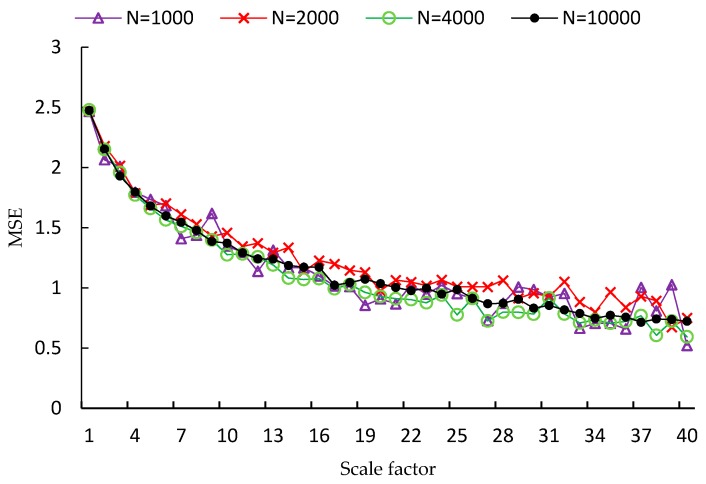
MSE values for white noise signals with different lengths.

**Figure 3:**
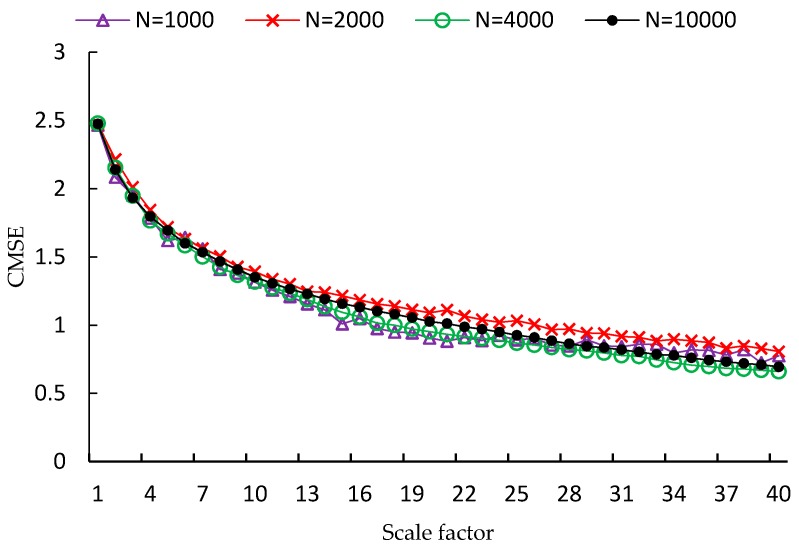
CMSE values for white noise signals with different lengths.

**Figure 4:**
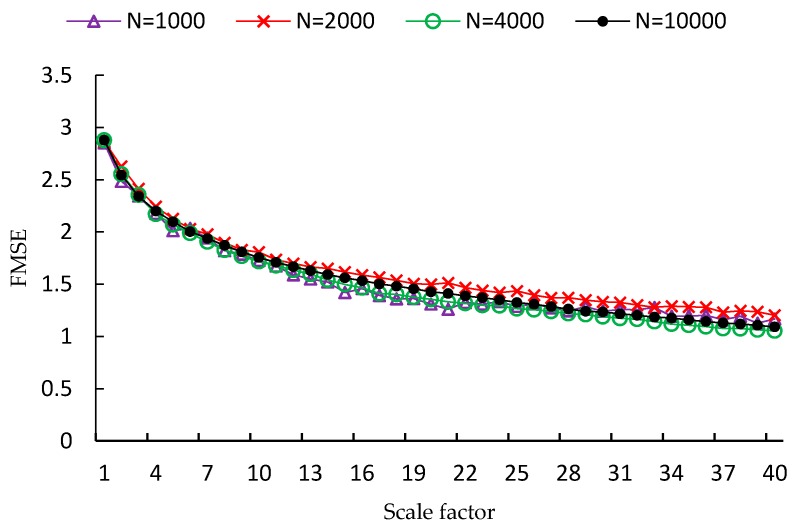
FMSE values for white noise signals with different lengths.

FMSE is first evaluated using two synthetic noise signals, including white noise and 1/f noise. Since the length of time series is a factor that influences the performance of MSE analysis, four different lengths of time series are used in the experiment, which are *N* = 1000, *N* = 2000, *N* = 4000, *N* = 10,000, respectively. For each type and each length of noise signal, one hundred independent time series samples are used to calculate the MSE, CMSE and FMSE values. Examples of MSE, CMSE and FMSE values for white noise signals are shown in Figure 2, Figure 3 and Figure 4, respectively.

From each of the three figures, it is easy to find that the curve of entropy values of white noise time series with more points is smoother than that with fewer points. This indicates that the variance of the entropy values increases as the length of time series decreases. By comparing the three figures, it is clear that the corresponding curve of MSE values is the most fluctuated among the curves of the three metrics. In fact, the FMSE has better stability than CMSE, although the improvement is not easily observed in the figures. For this reason, we will present more evidence of the improvement of FMSE over CMSE by comparing the coefficient of variation of entropy values calculated with FMSE and CMSE later.

**Figure 5:**
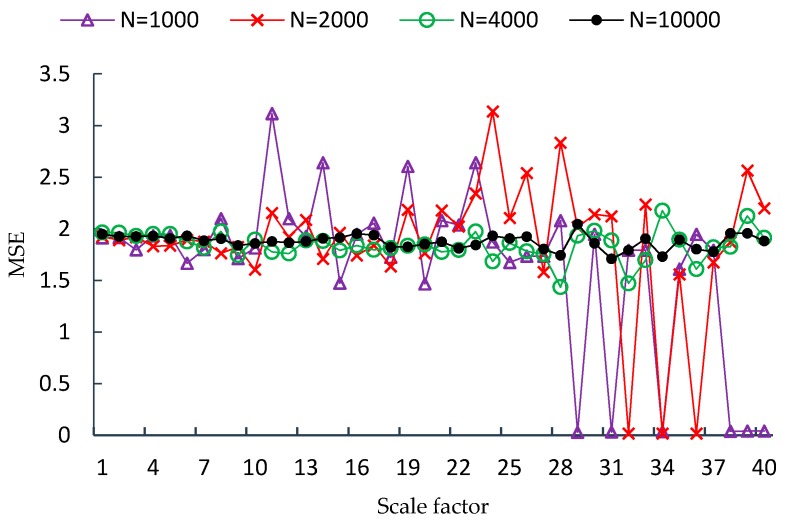
MSE values for 1/f noise signals with different lengths.

**Figure 6:**
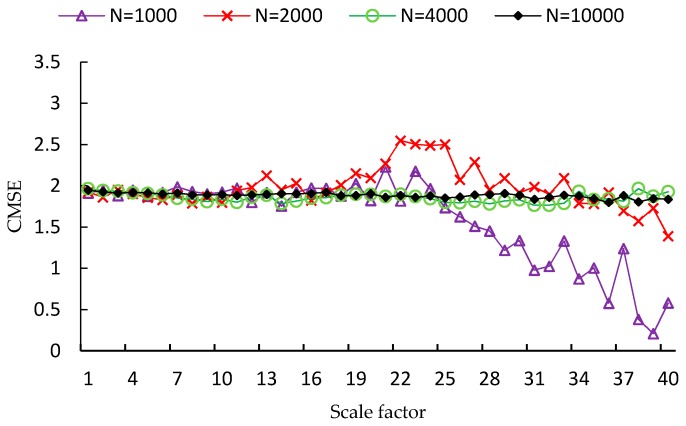
CMSE values for 1/f noise signals with different lengths.

**Figure 7:**
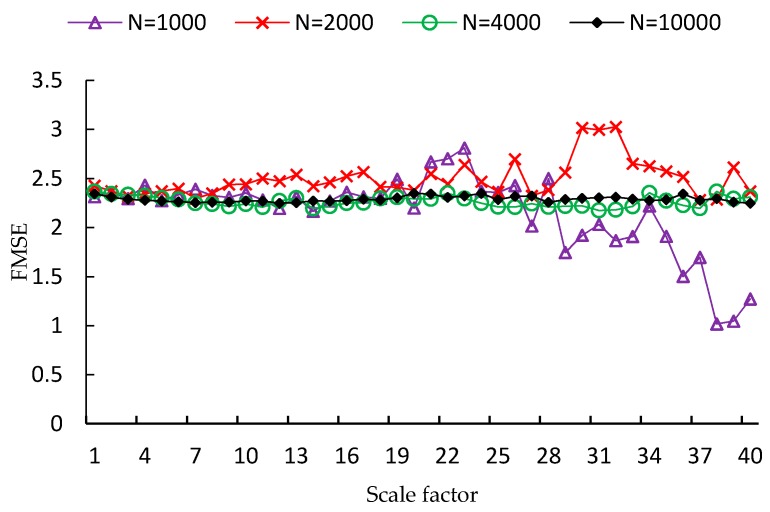
FMSE values for 1/f noise signals with different lengths.

Examples of MSE, CMSE and FMSE values for 1/f noise signals are shown in Figure 5, Figure 6 and Figure 7, respectively. Different from white noise, 1/f noise signal is time-correlated, so the entropy values of 1/f noise theoretically remain the same for different time scales.

From the figures, we find two similar trends. First, the curve of entropy values associated with more points is smoother than that associated with fewer points. This indicates that the variance of the entropy values increases as the length of time series decreases. Second, it is clear that the corresponding curve of MSE values is the most fluctuated among the curves of the three metrics. However, for large scales, it is clearer for 1/f noise than white noise that the FMSE shows better stability than CMSE. For the time series with 1000 points, when the scale factor is more than 20, MSE and CMSE have obvious large degrees of decline. On the contrary, there is no sustained increase or decrease, which is in line with the characteristics of 1/f noise sequence. Furthermore, the fluctuation of FMSE values is much smaller than that of MSE and CMSE values.

From the entropy curves of both the white noise and 1/f noise time series, we can see that the FMSE has a better performance than MSE and CMSE, especially when the time series is short. In other words, the FMSE has a better tolerant of short length of time series than the other two metrics. Hence, the FMSE is able to measure the complexity of time series more accurately than MSE and CMSE.

In the next, the convergence of the entropy values estimated from one hundred independent noise signals is examined. It is reasonable to assume that entropy values estimated for different samples generated by the same noise function should be convergent. In other words, the dispersion of the estimations for different samples from the same noise function is the lower the better. Since, the mean value of FMSE is different from that of MSE, CMSE, we use coefficient of variation instead of standard deviation to measure the convergence. The coefficient of variation (CV) is defined as the ratio of the standard deviation σ, over the mean μ:

(16) $$C_{v}=\frac{\sigma }{\mu }$$

**Figure 8:**
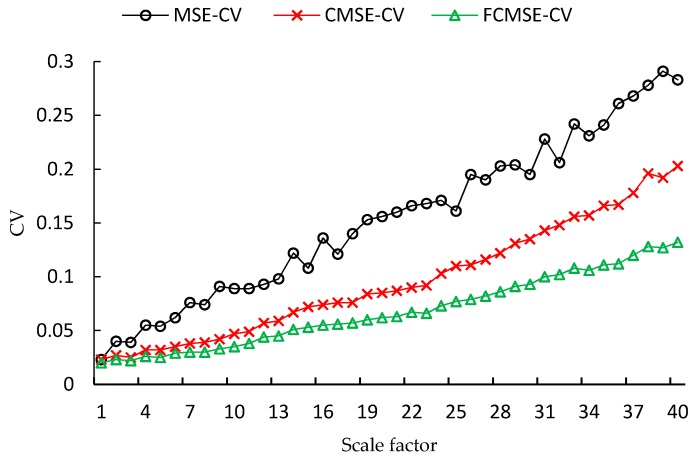
CVs of white noise with length 1000.

**Figure 9:**
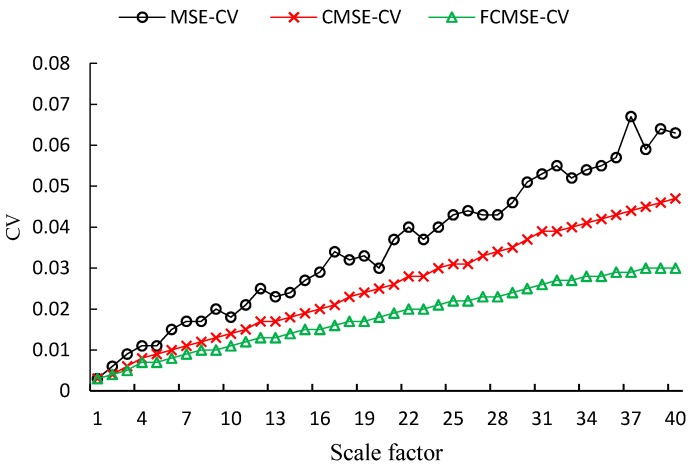
CVs of white noise with length 10,000.

The lower the CV of estimations for samples generated by the same noise, the better the performance is. The CVs of white noise with two different data lengths (*N* = 1000 and 10,000) are shown in Figure 8 and Figure 9, respectively. As shown in the figures, the CVs of estimations for time series with 1000 points are much larger than those of 10,000 points. It is obvious that the CVs of FMSE values are always the lowest among the three metrics. Furthermore, as the scale factor increases, the improvement in CV of FMSE over the other two becomes larger.

**Figure 10:**
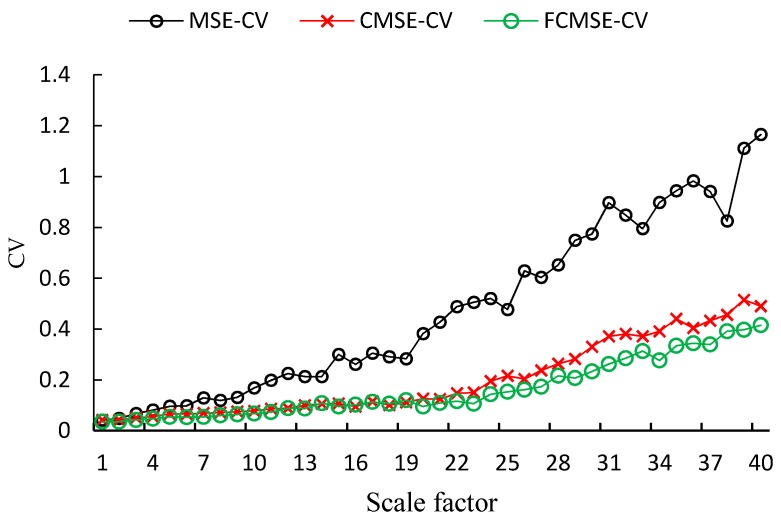
CVs of 1/f noise with length 1000.

**Figure 11:**
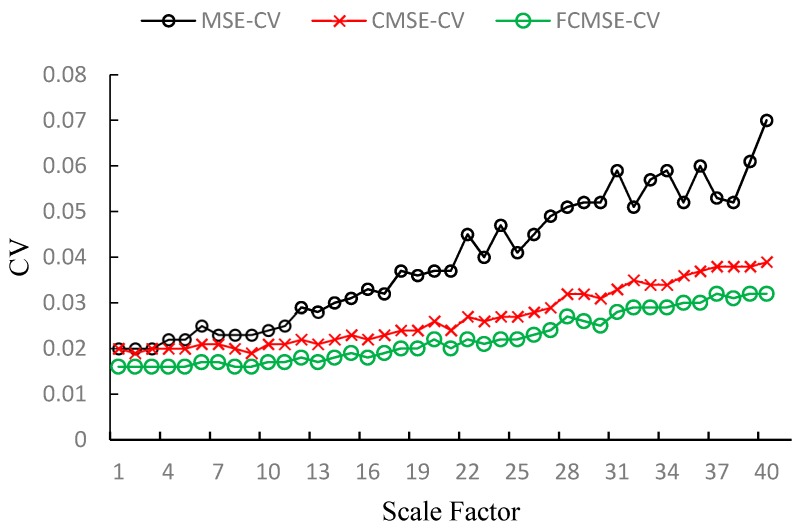
CVs of 1/f noise with length 10,000.

Figure 10 and Figure 11 show the CVs of 1/f noise with two different data lengths (*N* = 1000 and 10,000). From the two figures, it is found that the performance of FMSE is also better than the other two, but with a smaller improvement in comparison with that of white noise.

From the results for both white noise and 1/f noise, CVs of FMSE are smaller than those of the other two metrics. The decrease in CV becomes more significant when the scale factor increases. The improvement in CV indicates that the FMSE measures the complexity of time series with a higher stability and reliability than the other two metrics.

Table 1 provides the CVs of the three entropy metrics under different scale factors. As can be seen from the table, the CVs of the FMSE in each scale factor are smaller. For white noise signals with 1000, 2000, 4000 and 10,000 points, the aggregate improvement in CV of FMSE over MSE and CMSE is around 50% and 30%, respectively. For 1/f noise signals, the aggregate improvement in CV of FMSE over MSE is higher than 40%. In the three out of the four cases, the improvement of FMSE over CMSE is larger than 15%. For the 1/f noise that with 2000 points, the improvement is nearly 10%, which is also considerable. It is necessary to mention that, due to the limitation of space, we have not shown the CVs for all of the 40 scale factors in Table 1, but only six of them are shown.

### 5.2. Real Vibration Data

In this section, the FMSE is evaluated using real vibration data, which were obtained from the Case Western Reserve University (CWRU) Bearing Data Center [42]. The bearing equipment that generated vibration data was composed of two horsepower motors, a torque transducer, a dynamometer, and control electronics. The vibration data sets were collected with fault diameters of 7 mils (one mil is one thousandth of an inch) and the motor speeds was 1772 rpm. The bearing equipment include 6 conditions, which are normal states, ball faults, inner race faults and outer race faults located at 3, 6 and at 12 o’clock. The set of data was collected at a rate of 48,000 samples per second for drive end bearing faults.

In the evaluation, the vibration data is divided into 6 groups based on the 6 conditions. For each group, we divided the data into non-overlapping time series. After division, each group includes about 240 time series and each has a length of 2000. We calculated the mean of MSE, CMSE and FMSE values, respectively, for each group, and the scale factor is from 1 to 40.

**Figure 12:**
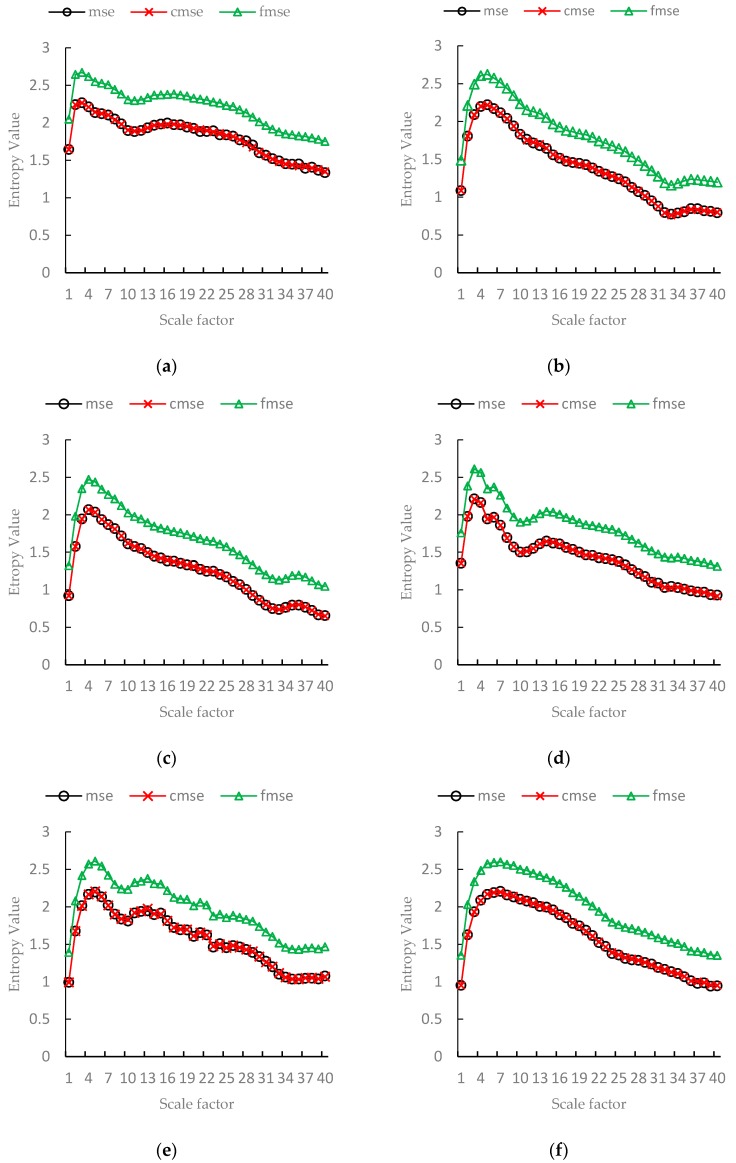
The means of MSE, CMSE, FMSE values on bearing vibration data (1730 rpm, 7 mils). (**a**) Normal state; (**b**) Outer race fault (3 o’clock position); (**c**) Outer race fault (6 o’clock position); (**d**) Outer race fault (12 o’clock position); (**e**) Ball fault; (**f**) Inner race fault.

The means of MSE, CMSE, FMSE values of vibration signals are shown in Figure 12. From Figure 12, we can see that the mean of MSE values is very close to that of CMSE. The mean of FMSE values is higher than that of MSE and CMSE, but the trend of FMSE is similar to MSE and CMSE. It means that FMSE can reflect the complexity change of time series completely.

**Figure 13:**
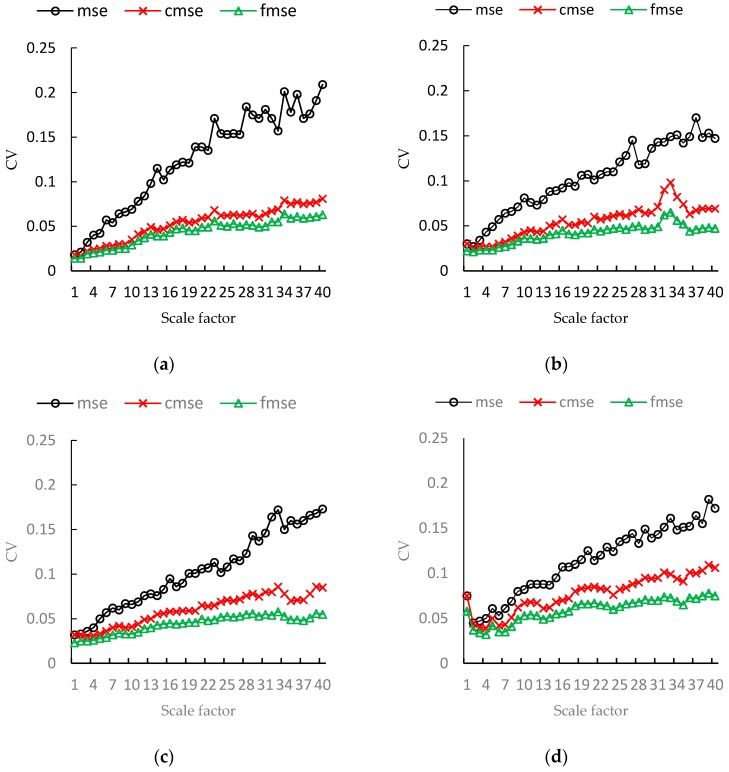

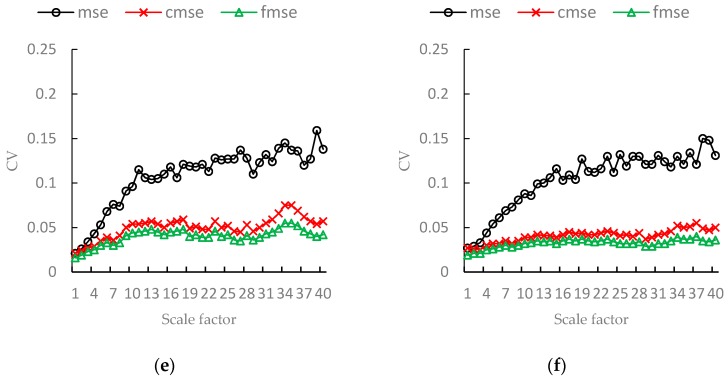
The CVs of MSE, CMSE, FMSE values on bearing vibration data (1730 rpm, 7 mils). (**a**) Normal state; (**b**) Outer race fault (3 o’clock position); (**c**) Outer race fault (6 o’clock position); (**d**) Outer race fault (12 o’clock position); (**e**) Ball fault; (**f**) Inner race fault.

It is also reasonable to assume that the entropy values of different samples generated by the same system with same condition should be convergent. Thus, we also use CV to evaluate the performance of MSE, CMSE and FMSE. Figure 13 and Table 2 shows the CVs of MSE, CMSE and FMSE for the vibration signals in all scale factors. We can see from Figure 13 that the CVs of FMSE values are smaller than that of MSE and CMSE values in every scale factor. The superiority of FMSE is especially significant in large scale factor. We provide the CVs for some scale factors in Table 2. In the table, column fault class lists the 6 different conditions. N means normal, B means ball fault, I means inner race fault. O3, O6 and O12 means outer race faults located at 3, 6 and 12 o’clock, respectively. The last column is the total decrease of CVs obtained by FMSE against MSE and CMSE for all scale factors. From the table, we can see that FMSE has lower CVs in all the scale factors at each condition of vibration signals. The total decrease in CVs of FMSE values against MSE and CMSE values reaches up to 68.87% and 26.87%, and at least 47.7% and 18.45%, respectively.

In this section, we evaluate the FMSE using synthetic noise signals and real vibration data. As can be seen, all the results show that FMSE has a lower coefficient of variation, or better stability and reliability, in measuring the structural complexity of time series, compared to MSE and CMSE.

## 6. Conclusions

Measurement of time series complexity and predictability is sometimes the cornerstone of applying time series analysis in solving topology and traffic control problems in sensor networks. In this paper, we have introduced the entropy metrics that are used to measure the complexity and predictability of time series. The existing entropy metrics become limited when the length of empirical time series is short. To this end, we propose the flexible multiscale entropy (FMSE) to measure the complexity of time series in this paper. The flexible multiscale entropy proposed in the paper introduces a new function for measuring and accumulating the similarity between time series patterns. The new accumulative function avoids the similarity of time series patterns changing suddenly between 0 and 1. The proposed flexible multiscale entropy is evaluated with both synthetic noise signals and real vibration data. The results show that flexible multiscale entropy has a better reliability and stability in measuring complexity of time series. The proposed method FMSE is useful for improving the performance of topology and traffic control techniques relied on time series analysis.

## 7. Future Work

Our work could be extended in several possible future work directions. The first direction is to go further to analyze time series generated by sensor networks with our proposed method. For example, investigate the spatiotemporal characteristics of time series in sensor networks, e.g., how time and space scales affect the behavioral patterns of sensor networks. Based on the understandings of spatiotemporal characteristics of sensor networks, we go further to build a situational awareness model for monitoring the running status of sensor networks. We will use this model to analyze behavior patterns of sensor networks, monitor and predict network connectivity, detect anomaly among sensor networks, and mitigate the congestion problem in sensor networks and so on. Furthermore, analyzing time series generated by sensor networks in real time and more energy efficiently is an important part of our future work as well.

## Figures and Tables

**Table 1:** CVs with different entropy and scale factor.

Data Length	Noise	Entropy	Scale Factor						Decrease in CV
			1	8	16	24	32	40	
1000	white noise	MSE	0.023	0.074	0.136	0.171	0.206	0.283	55.90%
		CMSE	0.023	0.039	0.074	0.103	0.148	0.203	29.28%
		FMSE	0.02	0.03	0.055	0.073	0.102	0.132	
	1/f noise	MSE	0.042	0.119	0.261	0.52	0.848	1.165	65.72%
		CMSE	0.042	0.071	0.095	0.195	0.381	0.49	19.57%
		FMSE	0.035	0.06	0.102	0.143	0.285	0.415	
2000	white nose	MSE	0.012	0.04	0.082	0.116	0.142	0.154	51.92%
		CMSE	0.012	0.03	0.051	0.074	0.096	0.116	29.01%
		FMSE	0.011	0.024	0.037	0.052	0.066	0.076	
	1/f noise	MSE	0.038	0.073	0.116	0.162	0.289	0.34	62.38%
		CMSE	0.038	0.052	0.065	0.081	0.095	0.089	9.42%
		FMSE	0.032	0.043	0.054	0.067	0.093	0.105	
4000	white noise	MSE	0.007	0.027	0.054	0.064	0.101	0.109	48.29%
		CMSE	0.007	0.02	0.036	0.053	0.072	0.084	29.89%
		FMSE	0.006	0.016	0.027	0.037	0.049	0.055	
	1/f noise	MSE	0.033	0.049	0.069	0.074	0.126	0.163	52.78%
		CMSE	0.033	0.035	0.042	0.052	0.056	0.066	16.79%
		FMSE	0.027	0.028	0.035	0.043	0.046	0.055	
10,000	white noise	MSE	0.003	0.017	0.029	0.04	0.055	0.063	48.37%
		CMSE	0.003	0.012	0.02	0.03	0.039	0.047	29.28%
		FMSE	0.003	0.01	0.015	0.021	0.027	0.03	
	1/f noise	MSE	0.02	0.023	0.033	0.047	0.051	0.07	43.74%
		CMSE	0.02	0.02	0.022	0.027	0.035	0.039	17.6%
		FMSE	0.016	0.016	0.018	0.022	0.029	0.032	

**Table 2:** The CVs with different entropy and scale factor of vibration signal.

Fault Class	Entropy	Scale						Decrease in CV
		1	8	16	24	32	40	
N	MSE	0.018	0.064	0.113	0.154	0.171	0.209	65.27%
	CMSE	0.018	0.03	0.051	0.062	0.067	0.081	18.45%
	FMSE	0.014	0.025	0.043	0.051	0.055	0.063	
O3	MSE	0.03	0.066	0.092	0.11	0.143	0.147	59.99%
	CMSE	0.03	0.036	0.057	0.061	0.09	0.069	24.87%
	FMSE	0.022	0.029	0.045	0.047	0.062	0.047	
O6	MSE	0.032	0.06	0.095	0.102	0.164	0.173	57.43%
	CMSE	0.032	0.042	0.058	0.069	0.08	0.085	26.32%
	FMSE	0.023	0.034	0.045	0.052	0.054	0.055	
O12	MSE	0.075	0.069	0.107	0.124	0.151	0.172	47.7%
	CMSE	0.075	0.051	0.07	0.076	0.101	0.106	23.1%
	FMSE	0.058	0.041	0.056	0.06	0.074	0.075	
B	MSE	0.021	0.074	0.118	0.126	0.124	0.138	62.71%
	CMSE	0.021	0.041	0.055	0.05	0.059	0.057	20.63%
	FMSE	0.016	0.033	0.045	0.04	0.045	0.042	
I	MSE	0.027	0.073	0.103	0.112	0.124	0.131	68.87%
	CMSE	0.027	0.032	0.042	0.044	0.043	0.05	20.92%
	FMSE	0.019	0.028	0.035	0.034	0.032	0.036	

## Appendix A. Analytical FMSE Results for White Noises

In this appendix, we provide the analytical derivations of FMSE for white noises with Gaussian distributions. In order to make this derivation possible, we assume linear Gaussian correlation in the white noises. In this appendix, we use *P*() for probability distributions and *p*() for probability density functions.

For the case *m* = 1, since there is no correlation between any data point and its preceding data points in white noise, FMSE equals the negative natural logarithm of the weighted probability that the distance between any two data points is less than or equal to *f*. Then, we have:

(A1) $$P_{f}(|y_{j}^{\tau }-y_{i}^{\tau }|\leq f)=\int_{-\infty }^{+\infty }\{\int_{y_{i}^{\tau }-f}^{y_{i}^{\tau }+f}p(y_{j}^{\tau })dy_{j}^{\tau }\}p(y_{i}^{\tau })dy_{i}^{\tau }-\int_{-\infty }^{+\infty }\left\{\frac{1}{f}\int_{y_{i}^{\tau }-f}^{y_{i}^{\tau }+f}p(y_{j}^{\tau })|y_{i}^{\tau }-y_{j}^{\tau }|dy_{j}^{\tau }\right\}p(y_{i}^{\tau })dy_{i}^{\tau }$$

Without loss of generality, we considered a white noise has a Gaussian distribution with a mean of zero and variance of σ. The coarse-grained white noise time series still has a mean of zero. However, the variance of decreases from σ to $\sigma_{\tau }$:

(A2) $$\sigma_{\tau }=\frac{\sigma }{\sqrt{\tau }}$$

where τ is the time scale, $\sigma_{\tau }$ represents the variance of the coarse-grained white noise time series. Then, we can derive the first part of the Equation (A1) is as follows:

(A3) $$\int_{-\infty }^{+\infty }\{\int_{y_{i}^{\tau }-f}^{y_{i}^{\tau }+f}p(y_{j}^{\tau })dy_{j}^{\tau }\}p(y_{i}^{\tau })dy_{i}^{\tau }\;=\frac{1}{2\sigma }\sqrt{\frac{\tau }{2\pi }}\int_{-\infty }^{+\infty }\left\{erf(\frac{t+f}{\sigma \sqrt{2/\tau }})-erf(\frac{t-f}{\sigma \sqrt{2/\tau }})\right\}e^{-t^{2}f/2\sigma^{2}}dt$$

Next, we will derive the second part of the Equation (A1). For the purpose of distinguishing symbols easier, we let t represents $y_{i}$, x represents $y_{j}$, then we have:

(A4) $$\int_{-\infty }^{+\infty }\left\{\frac{1}{f}\int_{y_{i}^{\tau }-f}^{y_{i}^{\tau }+f}p(y_{j}^{\tau })|y_{i}^{\tau }-y_{j}^{\tau }|dy_{j}^{\tau }\right\}p(y_{i}^{\tau })dy_{i}^{\tau }\;=\int_{-\infty }^{+\infty }\left\{\frac{1}{f}\int_{t-f}^{t+f}p(x)|t-x|dx\}p(t\right)dt\;=2\int_{-\infty }^{+\infty }\text{\{}\frac{1}{f}\int_{t}^{t+f}p(x)(x-t)dx\text{\}}p(t)dt\;=2\int_{-\infty }^{+\infty }\{\frac{1}{f}\int_{t}^{t+f}p(x)xdx\}p(t)dt-2\int_{-\infty }^{+\infty }\{\frac{1}{f}\int_{t}^{t+f}p(x)tdx\}p(t)dt\;=2\int_{-\infty }^{+\infty }\left\{\frac{1}{f}\frac{\sigma_{\tau }}{\sqrt{2\pi }}e^{-t^{2}/2\sigma_{\tau }^{2}}-\frac{1}{f}\frac{\sigma_{\tau }}{\sqrt{2\pi }}e^{-{\left(t+f\right)}^{2}/2\sigma_{\tau }^{2}}\right\}p(t)dt\;+2\int_{-\infty }^{+\infty }\frac{t}{2f}\{erf(t/\sigma_{\tau }\sqrt{2})-erf(\frac{t+f}{\sigma_{\tau }\sqrt{2}})\}p(t)dt\;=\frac{\sigma_{\tau }}{f\sqrt{\pi }}-\frac{\sigma_{\tau }}{f\sqrt{\pi }}e^{-f^{2}/4\sigma_{\tau }^{2}}+\frac{1}{f\cdot \sigma_{\tau }\sqrt{2\pi }}\int_{-\infty }^{+\infty }terf(t/\sigma_{\tau }\sqrt{2})\cdot e^{-t^{2}/2\sigma_{\tau }^{2}}dt\;-\frac{1}{f\sigma_{\tau }\sqrt{2\pi }}\int_{-\infty }^{+\infty }terf(\frac{t+f}{\sigma_{\tau }\sqrt{2}})e^{-t^{2}/2\sigma_{\tau }^{2}}dt$$

Equations (A3) and (A4) can be approximated numerically. For example, we can set the following conditions for numerical calculation: (1) dt→Δt=1/5000; (2) the range of the calculation is [−3,3]=[−(N/2)Δt, (N/2)Δt], with N= 30,000. The values calculated from above analytical equations are in good agreement with those obtained by the FMSE algorithm on simulated white noise time series. It is necessary to mention that the derivation of FMSE values is similar to that of MSE, we would like to refer readers to reference [6] for more detailed information.
